# Setting global research priorities for child protection in humanitarian action: Results from an adapted CHNRI exercise

**DOI:** 10.1371/journal.pone.0202570

**Published:** 2018-08-22

**Authors:** Laura Gauer Bermudez, Katharine Williamson, Lindsay Stark

**Affiliations:** 1 Columbia University School of Social Work, New York, New York, United States of America; 2 Save the Children, London, United Kingdom; 3 Department of Population and Family Health, Columbia University Mailman School of Public Health, New York, New York, United States of America; 4 George Warren Brown School of Social Work, Washington University in Saint Louis, St. Louis, Missouri, United States of America; Johns Hopkins School of Public Health, UNITED STATES

## Abstract

**Background:**

Armed conflict, natural disaster, and forced displacement affect millions of children each year. Such humanitarian crises increase the risk of family separation, erode existing support networks, and often result in economic loss, increasing children’s vulnerability to violence, exploitation, neglect, and abuse. Research is needed to understand these risks and vulnerabilities and guide donor investment towards the most effective interventions for improving the well-being of children in humanitarian contexts.

**Methods:**

The Assessment, Measurement & Evidence (AME) Working Group of the Alliance for Child Protection in Humanitarian Action (ACPHA) identified experts to participate in a research priority setting exercise adapted from the Child Health and Nutrition Research Initiative (CHNRI). Experts individually identified key areas for research investment which were subsequently ranked by participants using a Likert scale. Research Priority Scores (RPS) and Average Expert Agreement (AEA) were calculated for each identified research topic, the top fifteen of which are presented within this paper.

**Results:**

Intervention research, which aims to rigorously evaluate the effectiveness of standard child protection activities in humanitarian settings, ranked highly. Child labor was a key area of sector research with two of the top ten priorities examining the practice. Respondents also prioritized research efforts to understand how best to bridge humanitarian and development efforts for child protection as well as identifying most effective way to build the capacity of local systems in order to sustain child protection gains after a crisis.

**Conclusions:**

Rigorous, scientific research that assesses the scope of child protection risks, examines the effectiveness of interventions to improve child well-being, and translates evidence to practice is critical. Findings from this research priority setting exercise offer guidance for a global research agenda on child protection in humanitarian settings, encouraging cooperation among donors, implementers, and academics to pursue a coordinated approach to evidence generation.

## Introduction

The number of people affected by humanitarian crises is on the rise, perpetuated by armed conflict and natural disasters [1]. In 2017, there were over 65 million forcibly displaced people, over half of whom were under the age of 18 [2]. In addition, over one billion children live in countries affected by armed conflict [3]. Environmental factors, including climate change, are likely to increase the number of conflicts and intensify the severity of natural disasters [4–5]. Armed conflicts and large-scale disasters increase the potential for family separation and the erosion of existing support systems, putting children at risk of abuse, exploitation, violence, and neglect. The widespread economic shocks that often accompany humanitarian crises create further vulnerabilities for children when households employ negative coping strategies to manage economic stress. In Lebanon, where over one million Syrian refugees have been registered with the United Nations High Commissioner for Refugees (UNHCR), child marriage and child labor have been reported as families struggle financially [6–7]. Children in circumstances of economic and physical insecurity are also at risk of child trafficking, sexual exploitation, and recruitment by armed forces and extremist groups.

Within these contexts, child protection experts in non-governmental organizations (NGO), multilateral institutions such as the UN Children’s Fund and the United Nations High Commissioner for Refugees, work to prevent and respond to incidents of abuse, neglect, exploitation, and violence against children. These efforts can take the form of broader systems-strengthening interventions that seek to build the capacity of national actors to implement effective social support systems that care for children and families, both in formal and informal spheres. As a complement to systems strengthening, child protection initiatives may also take the form of direct implementation, such as the establishment of “Child Friendly Spaces (CFS)” that allow children safe zones to play, parenting trainings that emphasize alternatives to physical punishment, or family tracing and reunification for unaccompanied or separated children. Yet, the assumptions that drive such child protection efforts in humanitarian practice have not yet been fully based on scientific evidence. Protection risks are often estimated and prioritized based on anecdotal accounts [8], definitions of child protection concepts are often not standardized [9], and there is scant evidence on the effectiveness of many of the sector’s universally agreed upon standard interventions [10–12].

To begin addressing these gaps in empirical research within the sector of child protection in humanitarian contexts, a research priority setting exercise, adapted from the Child Health and Nutrition Research Initiative (CHNRI), was undertaken to identify and rank research priorities. This manuscript presents the process and results of this participatory ranking methodology designed to guide future research investment.

## Methods

**Fig 1:**
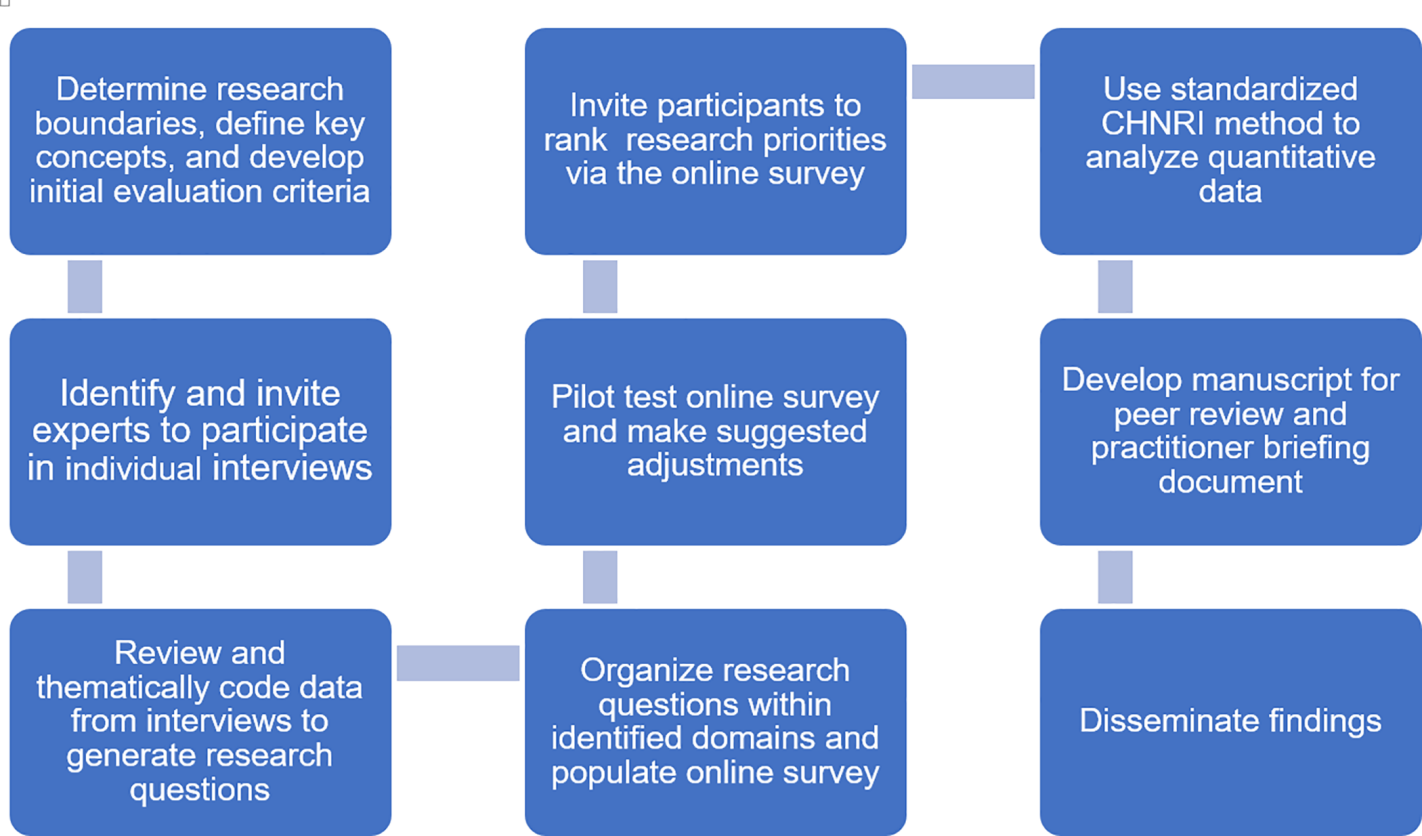
Research priorities setting exercise flow diagram.

The Child Health and Nutrition Research Initiative (CHNRI) was designed as a tool to help guide policy and investment in global health research, specifically children’s health. CHNRI has since been used to establish research priorities across a broad array of global health disciplines [13–20]. The method is comprised of four stages (i) determining the boundaries of investigation and creating evaluation criteria; (ii) obtaining and systematically listing input from key stakeholders on critical priorities/tasks (referred to as “research questions”) to address gaps in sectoral evidence or knowledge; (iii) enlisting stakeholders to rank the research questions based on a pre-defined set of evaluation criteria; (iv) calculation of research priority scores and agreement between experts (Fig 1]. A more detailed explanation of the CHNRI method has been published elsewhere [21–23].

The present study was commissioned by the Assessment, Measurement and Evidence Working Group of the Alliance for Child Protecton in Humanitarian Action (ACPHA) and was informed by prior consensus-building efforts in the sector [24–25]. In collaboration with a Lead Researcher, the CHNRI method was adapted to prioritize research topics in the sector of child protection in humanitarian settings. For the purposes of this exercise, a ‘humanitarian setting’ was defined as “acute or chronic situations of conflict, war or civil disturbance, natural disaster, food insecurity or other crises that affect large civilian populations and result in significant excess mortality” [26]. The goal of ‘child protection’ efforts are “to protect children from abuse, neglect, exploitation, and violence” [27]. And ‘children’ were defined as “individuals under the age of 18” [28].

Experts working on issues of child protection in humanitarian settings were then invited to take part in semi-structured interviews to discuss the gaps in knowledge and evidence that existed within the sector and to generate research priorities to address these gaps. Forty-seven experts participated in this first round of evidence generation with representatives from Non-Governmental Organizations (NGOs), United Nations (UN) agencies, donor agencies, and research institutions. Experts were initially identified through three coordination bodies–the Alliance for Child Protection in Humanitarian Action (ACPHA), the Child Protection Area of Responsibility (CP AoR), and UNHCR with the network extended through snowball sampling. Respondents were strategically diversified to include inputs from those involved in various child protection job functions including implementation, coordination, policy development, and academia from a range of geographic locations (Table 1]. Recruitment continued on a rolling basis and ended once data saturation, defined as the point at which no new data were being generated, was achieved. The final sample was consistent with previous research that identified 45–55 as the number of experts at which collective opinion stabilizes [29].

**Table 1:** Descriptive characteristics of first round respondents (N = 47).

n (%)	
***Location***	
Field-Based	19 (40.4)
Non-Operational Setting	28 (59.6)
Geographic Region of Focus*	
Global	26 (55.3)
Middle East & North Africa	6 (12.8)
West & Central Africa	6 (12.8)
East & Southern Africa	7 (14.9)
Latin America & the Caribbean	1 (2.1)
South & Southeast Asia	4 (8.5)
Europe	1 (2.1)
Job Function*	
Implementation/Operations	
Technical Advice	15 (31.9)
Coordination	45 (95.7)
Policy	31 (66.0)
Academic Research	20 (42.6)
Program Evaluation	11 (23.4)

*Respondents were allowed to answer all that apply

Aligned with prior CHNRI studies in humanitarian contexts [14], interviews were held via Skype with experts notified in advance that they would be requested to provide their opinions on the most important areas for investment to improve the state of evidence in the field of child protection in humanitarian settings in the next 3–5 years. Participants were encouraged to follow up by email in the event they were able to generate further ideas after the interview had concluded.

Through an iterative process, the Lead Researcher then collated 24 hours of interview notes to identify 90 unique research priorities, condensing interrelated research ideas and simplifying concepts for use in the ranking exercise. The priorities were then thematically organized into the following pre-determined themes—Epidemiological Research; Policy and Systems Research; and Intervention Research (Table 2]. The research team provided review and consensus on the themes and categorization after which the areas for research were listed within the online survey. The survey was pilot tested by individuals who were not involved in the development of research questions but who had general knowledge of humanitarian concepts and survey design. Further, to ensure that question order did not bias results, we implemented a page randomization that shuffled page order within the survey for each new respondent.

**Table 2:** Research priorities by theme.

Research Type	Abbreviation	Number of Items
Basic epidemiological and social science research which aims to define the incidence or prevalence of abuse, exploitation, or violence against children or identify the underlying risk factors associated with violations against children.	EPI	22
Sub-Theme #1 –Measuring the incidence or prevalence of child protection concerns in humanitarian settings		13
Sub-Theme #2 –Understanding risk and resilience		9
Policy and systems research which seek to improve the efficiency of child protection systems to deliver effective interventions.	PSR	11
Sub-Theme #1 –Examining a policy or system’s capacity to reduce abuse, exploitation, or violence against children		6
Sub-Theme #2 –Examining a policy or system’s capacity to deliver effective interventions		5
Research to improve existing interventions or develop new interventions.	INT	57
Sub-Theme #1 –Evaluating existing interventions		22
Sub-Theme #2 –Improving the implementation of interventions		12
Sub-Theme #3—Improving the design of interventions		10
Sub-Theme #4 –Improving structures to facilitate data collection and analysis		13

Experts who participated in the interview process were invited to take part in the online ranking portion of the prioritizatio exercise. Two additional experts who were either not previously available or who reached out to participate after the period for interviews had passed, were also invited to take part in survey.

Each of the 90 research priorities were ranked on four criteria: (i) Relevance–research will support learning that contributes to the prevention and response to abuse, neglect, exploitation, or violence in humanitarian settings; (ii) Feasibility–research is feasible to conduct in an ethical way; (iii) Originality–research will generate new findings or methods; and (iv) Applicability–research will be readily applied to programs and policies. Relative weights were not assigned to scoring criteria. For each research question, participants were offered six possible responses: strongly agree (5 points); agree (4 points); undecided (3 points); disagree (2 points); strong disagree (1 point); and insufficiently informed (considered non-applicable/no response). The scoring matrix was a deviation from past CHNRI studies which typically offered four possible responses–yes (1 point), no (0 points), undecided (0.5 points), and insufficiently informed/no response. In the development of the present research design, the study team elected to use a full Likert scale to allow for greater granularity when analyzing scores.

Aligned with the CHNRI methodology [13–20], every research question was provided a priority score under each of the four judging criterion, calculated by taking the point totals and dividing them by the maximum number of points available, after excluding from the denominator those who did not answer the question or reported they were insufficiently informed, a percentage was calculated [14]. For each question, the overall Research Priority Score (RPS) was then calculated by taking the mean of the total priority scores for each judging criterion, as calculated above. Research questions were then ranked from highest to lowest on overall priority scores and the top fifteen presented in Table 3. Standard deviations for RPS are also included to show the variation between total priority scores for each judging criterion (Table 3, S1 Annex).

**Table 3:** Top fifteen research priorities based on research priority score.

Overall Rank	Research Question	Research Type	Relevance	Feasibility	Originality	Applicability	Average Expert Agreement AEA	Research Priority Score RPS (SD)
**1**	Rigorously evaluate the effectiveness of cash-based social safety nets to improve child well-being	INT	90.29	87.27	80.00	87.74	85.63	86.33 (4.42)
**2**	Rigorously evaluate the effectiveness of family strengthening interventions to improve child well-being	INT	89.44	83.03	79.35	82.94	83.09	83.69 (4.20)
**3**	Identify best practices for para-social work models in humanitarian settings	PSR	86.45	84.67	77.93	82.67	78.21	82.93 (3.67)
**4**	Rigorously evaluate the effect of multi-sectoral programs on child well-being. Analyze how various components interact with one another.	INT	86.06	78.62	85.16	81.29	83.63	82.78 (3.46)
**5**	Examine systems strengthening interventions to determine which have a measurable impact on children.	PSR	89.47	79.44	77.22	83.33	77.27	82.37 (5.37)
**6**	Estimate the prevalence of child labor in humanitarian settings	EPI	88.95	81.11	76.00	82.78	80.45	82.21 (5.34)
**7**	Identify best practices for bridging humanitarian and development initiatives for CP systems strengthening	PSR	84.44	84.00	76.97	82.86	75.38	82.07 (3.46)
**8**	Rigorously evaluate the effectiveness of psychosocial programming to improve child well-being	INT	88.33	86.06	70.00	82.35	78.02	81.69 (8.17)
**9**	Rigorously evaluate the added value of child protection interventions when mainstreamed within other sectors (i.e. health, education)	INT	87.50	76.43	82.07	79.33	82.88	81.33 (4.71)
**10**	Rigorously evaluate the effectiveness of interventions to reduce child labor	INT	86.47	81.94	76.13	80.63	85.75	81.29 (4.26)
**11**	Rigorously evaluate the effectiveness of capacity building interventions (for gov’t officials, social service workers, para social workers) to understand their impact on child well-being	PSR	85.95	77.65	77.14	83.43	80.68	81.04 (4.34)
**12**	Identify risk factors associated with children with disabilities (with a particular emphasis on non-observable disabilities)	EPI	84.12	80.63	78.06	81.29	81.91	81.02 (2.49)
**13**	Rigorously evaluate the effectiveness of case management to improve child well-being	INT	86.47	81.82	75.76	80.00	73.62	81.01 (4.44)
**14**	Identify best practices for engaging the local social service workforce in emergency settings	PSR	84.74	81.08	77.06	81.11	76.29	81.00 (3.14)
**15**	Translate existing literature on humanitarian emergencies in urban settings to better adapt CP program to current contexts	INT	86.25	80.00	76.55	80.00	77.22	80.70 (4.04)

In addition, the Average Expert Agreement (AEA) was calculated for each research question. In order to obtain AEA values, we consolidated “strongly agree” and “agree” as well as “strongly disagree” and “disagree”. For each judging criterion, the number of modal responses was then divided by the total number of scorers for that question, again excluding those who did not answer the question or who reported they were insufficiently informed on the research question being assessed. Following this calculation, the ratios were then summed and divided by the number of judging criteria.

Both RPS and AEA were calculated for the entire group of respondents as well as for sub-groups, in order to analyze differences in priorities for those located in field settings as compared to those based in non-operational settings. Data were analyzed using Microsoft Excel.

### Ethics statement

Formal ethics review is usually not requested for undertaking CHNRI exercises [13–20] as the exercise does not involve personal or otherwise sensitive data. Participants were solicited via established professional networks whose purpose is to facilitate and enable information-sharing. Prior to participation in initial Skype interviews, all participants were informed on the nature of the research and the anonymity of their feedback.

## Results

Of the 49 respondents invited to take part in the online ranking, 41 experts participated, eliciting a response rate of 83.7 percent. Research questions from all three of the research domains (epidemiological research; policy and systems research; and intervention research) featured in the top 15 research priorities. Intervention research was the most predominant domain voted upon by experts with 8 of the top 15 priorities identified falling within this realm. Policy and systems research followed with 5 priorities and epidemiologic research with only 2 featured priorities ranking in the top 15 (Table 3).

The range of overall RPS was 63.28 to 86.33, with the highest ranked priority being the rigorous evaluation of the effectiveness of cash-based social safety nets to improve child well-being. Within the top 15 priorities, RPS ranged from 80.70 to 86.33. Intervention research which aims to rigorously evaluate the effectiveness of standard child protection activities provided in humanitarian settings ranked highly. Two questions concerning child labor, specifically estimating the prevalence and understanding the effectiveness of interventions to reduce the practice, ranked in the top ten priorities. Respondents also prioritized research efforts to understand how best to mobilize local systems, including the local social service workforce and para-social work models, in order to sustain child protection gains after international actors have departed a crisis.

**Fig 2:**
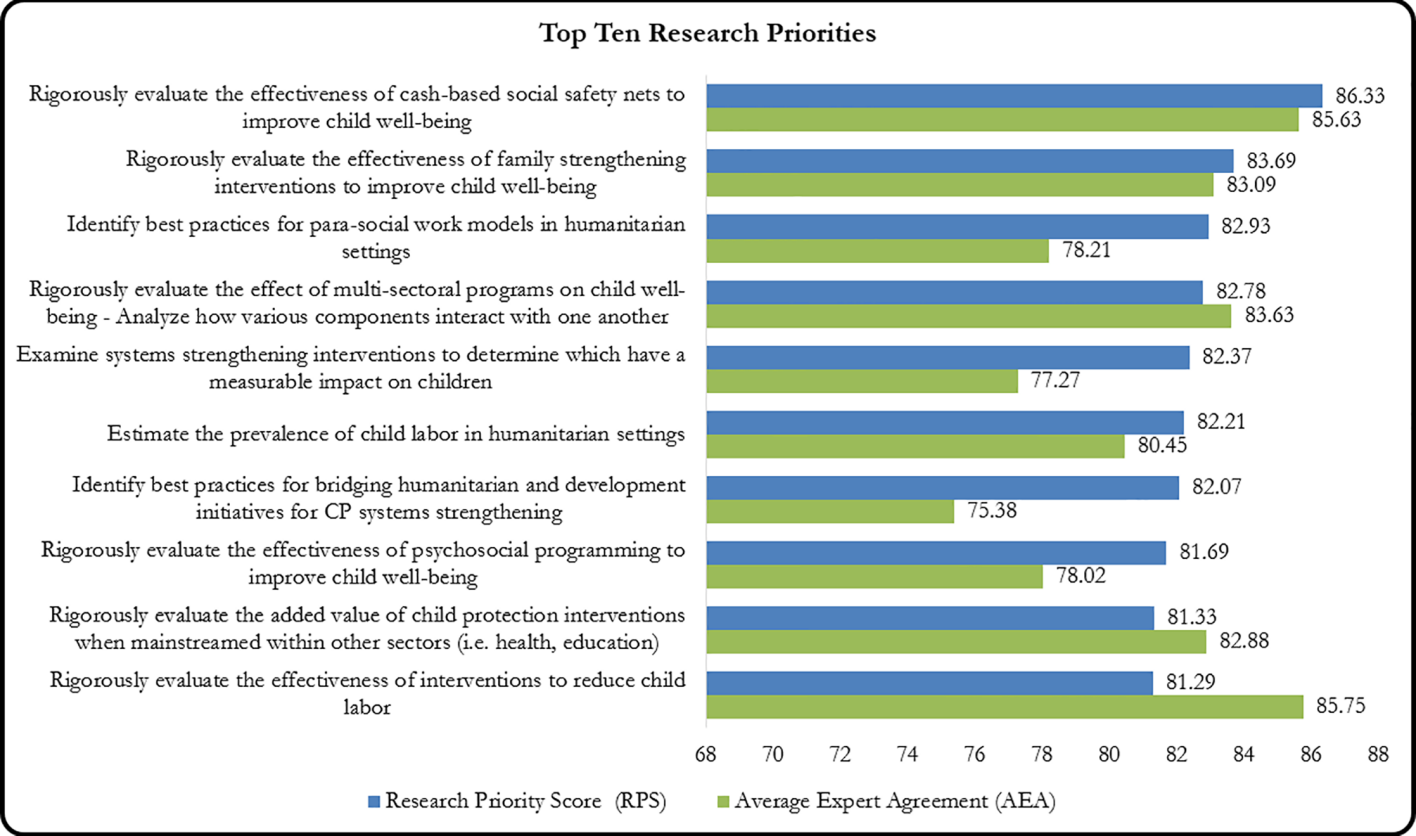
Top ten research priorities comparing RPS & AEA.

AEA scores ranged from 41.55 to 85.63, representing the percentage of respondents who provided the same score on a research priority (averaged across four judging criteria). For the top 15 research investment options, AEA ranged from 69.04 (to build the capacity of child protection sector staff in empirical research design and data analysis planning) to 85.75 (to evaluate the effectiveness of interventions to reduce child labor) (Table 3). We found higher levels of respondent agreement among research questions with higher RPS rankings, demonstrating that a certain level of consensus was attained in order for research topics to be prioritized in the higher ranks (Fig 2).

Standard deviations (SD) were also analyzed in order to assess variation between the judging criterion. Among the top 15 reserch priorities, SDs ranged between 2.5 and 5.4 with the exception of the evaluation of psychosocial programming with an SD of 8.2 due to the comparatively lower score provided on Originality. This is likely due to the recent work on this particular topic that has been widely circulated [30] and therefore was deemed less original in the ranking process.

When comparing all RPS scores among respondents who resided within an operational setting versus those who did not, there was a correlation co-efficient of 0.32, indicating a weak but positive association. The top ten research priorities differed between the two groups (Table 4). With the exception of rigorously evaluating family strengthening programs, which ranked highly for both groups of respondents, there were no other priorities that jointly ranked among the top ten. For field-based respondents, the most important initiative was to identify best practices for bridging humanitarian and development initiatives for child protection system strengthening. Field-based respondents tended towards the identification of best practices while also prioritizing capacity building for child protection sector staff in empirical research design and data analysis planning. In contrast, respondents who were not based in operational settings showed greater enthusiasm for the rigorous evaluation of interventions, with an examination of the effects of cash-based social safety nets on child well-being outcomes ranking highest.

**Table 4:** Top 10 research priority scores between field and non-field based respondents.

Overall Rank	Research Question	Research Type	Research Priority Score (RPS)
**Field-Based Respondents**			
**1**	Identify best practices for bridging humanitarian and development initiatives for CP systems strengthening	PSR	87.59
**2**	Identify best practices for holistic/integrated case management in emergency contexts	INT	87.50
**3**	Estimate the prevalence of child labor in humanitarian settings	EPI	87.48
**4**	Identify best practices for para-social work models in humanitarian settings	PSR	87.47
**5**	Rigorously evaluate the added value of child protection interventions when mainstreamed within other sectors (i.e. health, education)	INT	87.29
**6**	Identify best practices for operationalizing CP within the PSS pyramid	INT	86.89
**7**	Rigorously evaluate the effectiveness of family strengthening interventions to improve child well-being	INT	86.89
**8**	Build the capacity of the child protection sector staff in empirical research design and data analysis planning	INT	86.79
**9**	Identify best practices for sustainable, long-lasting engagement on interventions such as child protection committees	INT	86.12
**10**	Estimate the prevalence of children in alternative care	EPI	85.85
**Non-Field Based Respondents**			
**1**	Rigorously evaluate the effectiveness of cash-based social safety nets to improve child well-being	INT	88.44
**2**	Rigorously evaluate the effectiveness of interventions to reduce child labor	INT	83.78
**3**	Rigorously evaluate the effect of multi-sectoral programs on child well-being. Analyze how various components interact with one another.	INT	83.29
**4**	Identify best practices for engaging the local social service workforce in emergency settings	PSR	83,09
**5**	Identify risk factors associated with children with disabilities (with a particular emphasis on non-observable disabilities)	EPI	81.61
**6**	Rigorously evaluate the effectiveness of family strengthening interventions to improve child well-being	INT	81.08
**7**	Rigorously evaluate the effectiveness of capacity building interventions (for gov’t officials, social service workers, para social workers) to understand their impact on child well-being	PSR	81.00
**8**	Examine systems strengthening interventions to determine which have a measurable impact on children.	PSR	80.85
**9**	Translate existing literature on social-ecological models into program design	INT	80.56
**10**	Rigorously evaluate the effectiveness of psychosocial programming to improve child well-being	INT	80.54

## Discussion

The limitations to rigorous research on child protection in humanitarian crises are notable, with harsh operational conditions, short project cycles, and inadequate funding all considered hindrances to scientific inquiry on child protection within these contexts [31–32]. However, recent efforts have begun to demonstrate that robust social science methodologies within the sector are both needed and possible [33–35]. This prioritization exercise, which is among the first known systematic inquiries on research investments for child protection in humanitarian contexts using the CHNRI methodology, offers initial insight on the research interests and evidence needs of sector experts.

Intervention research comprised three of the top four research priorities, aligning with many previous CHNRI studies that have similarly found intervention research to be of importance to stakeholders [36]. As previously noted, there is a dearth of rigorous evaluation to determine the effectiveness of common child protection interventions in humanitarian settings. The lack of quantitative data to document intervention effectiveness inhibits the ability of humanitarian actors to design evidence-based programs, a hindrance increasingly problematic for funding appeals and policy advocacy. This prioritization suggests that understanding intervention effectiveness is of particular interest to the sector, ranging from examinations of family-strengthening to capacity-building interventions to activities aimed at reducing child labor. Because the sample more heavily represents individuals in technical advisory and other operational capacities, the interest in intervention research most visibly highlights the needs of practitioners to have their programming rigorously tested and evaluated with respect to child well-being outcomes.

As the top priority among both intervention research topics and the entire ranking exercise, understanding the effects of cash-based social safety nets on child well-being outcomes has emerged as highly importance for the sector. Cash transfers have gained prominence as multiple studies have found them effective in improving the welfare of children, including through improved health and nutrition outcomes as well as increased educational attainment [37–39]. The assumption driving the proliferation of cash-based social safety net interventions in humanitarian contexts is that they are an effective way of mitigating crisis-induced economic shocks, thereby preventing the use of coping strategies that may have negative effects on children such as school drop-out, child labor, and family separation. Yet, these assumptions have not been fully tested within disaster, conflict-affected, or displacement contexts, environments where children face unique risks and vulnerabilities. Further, the majority of existing evidence on the effects of cash transfers do not examine child protection outcomes such as reductions in violence, abuse, and exploitation, information of great interest to sector experts.

In addition to understanding the effectiveness of singular child protection interventions on child-well-being outcomes, experts indicated a need to also evaluate multi-sectoral interventions, considering this one of the highest priorities for research. A relatively broad mandate, this methodological research priority underscores the need for study designs that allow for the rigorous evaluation of multiple components within increasingly complex program designs, including analyses on how various components interact with one another. Such research endeavors are inherently more complicated, yet recent guidance from the global health sector has shown this to be a priority that spans disciplines within development and humanitarian assistance [40–42].

Similarly, as multi-sectoral and interdisciplinary interventions are prioritized by funders, experts within this study have identified a need to quantitatively demonstrate the added value of child protection interventions when mainstreamed within other sectors, such as health, nutrition, or education. Prior research on the effects of nutrition supplementation and play/stimulation on stunted children in Jamaica provides an example of how social scientists have captured the additive effects of non-sector related interventions [43]. If protection interventions are found to be effective in improving non-protection related outcomes for children, this type of evidence would support an argument that child protection considerations and/or program components are necessary to achieve desired results in other areas of humanitarian relief.

Child labor in humanitarian settings was also a common theme with both intervention effectiveness and prevalence data among the top 10 priorities for research investment. Similar to cash transfers, child labor has been examined across multiple development settings [44–46], however, data from humanitarian contexts is extremely sparse and generally limited to anecdotal information. As urban environments have become a more common setting for humanitarian crises, there is an increased risk that children will be used for begging, street vending, and other forms of exploitation [47–48]. There is a need to understand the prevalence, dynamics, and effective interventions to reduce this protection risk for children who have been displaced as well as children from affected host communities.

In order for child protection programming to be more responsive to current humanitarian contexts, experts felt that there was value in 1) better understanding the protection risks of children with disabilities (particularly non-observable disabilities) and 2) translating any existing evidence on implementing humanitarian programs in urban settings into more tangible guidance for CP practitioners. Disability inclusion has gained traction as a critical component within humanitarian assistance, however, experts noted this work to primarily address physical disabilities where programmatic accommodations are often tangible and straightforward, such as the fitting and distribution of assistive devices. In contrast, many experts noted feeling ill-equipped to properly serve children with cognitive and intellectual disabilities, agreeing that an examination of the protection risks for children with disabilities, particularly non-observable disabilities, should be prioritized.

Similarly, experts felt more guidance on child protection programming in urban humanitarian crises would be beneficial. Indeed, as rapid urbanization has resulted in more densely population cities and towns, the potential impacts of a humanitarian crisis increase, particularly in areas with weak infrastructure and insufficient governance [49]. The Syrian refugee crisis has seen over 5 million people flee to neighboring countries, seeking refuge predominately in the cities and towns of Lebanon and Jordan with another 6 million internally displaced within Syria, again primarily in urban and peri-urban settings [50]. This trend differs from past decades of humanitarian assistance that was largely provided within camp-based settings, requiring a new framework for understanding how best to support children in crisis. Other actors within humanitarian response have begun to give this issue greater attention in the past several years [51–52] enabling the identified priority of secondary literature review and as relevant, the translation and integration of evidence into child protection strategies and program design.

Localization and sustainability were also key themes. Within the top 15 research priorities, experts conveyed a need to identify best practices for both engaging the local social service workforce in emergency settings and establishing sustainable para-social work models such that structures will exist past the duration of humanitarian intervention. At the same time, respondents would like to understand best practices for bridging humanitarian and development initiatives for child protection systems strengthening. Taken together, these items demonstrate a desire to understand how best to engage local social service structures (formal and informal) and connect the work done during a crisis to a longer-term development agenda.

When scrutinizing the findings further, three trends emerged. First, among the top 15 research priorities, participants routinely scored research questions much higher for relevance than originality. It is speculated that this score variation may be a result of recent efforts by the sector to discuss and advocate for a more robust evidence base in humanitarian contexts [53–55]. The relatively frequent discussion about these evidence needs may have made a number of research questions appear unoriginal to participants yet still highly relevant because the research had yet to be carried out. This finding highlights the readiness of child protection experts to move forward an actionable research agenda for humanitarian settings.

Next, there were notable differences in the priorities of field and non-field based staff with only one research topic ranking within the top ten for both sub-groups (rigorously evaluate the effectiveness of family strengthening interventions to improve child well-being). As compared to non-field based respondents, those residing within an operational setting were less likely to identify rigorous evaluation within their top priorities. Instead, these respondents tended towards the identification of best practices, a logical reaction given that such research would presumably result in straight-forward guidance to program design. At the same time, field-based staff highly ranked capacity building in empirical research design and data analysis planning for the child protection sector, demonstrating a desire to build the skills required to further evidence generation.

Lastly, our study explored research topics within the professional sector of “child protection in humanitarian settings”, which had a rather expansive purview. As such, some of the research priorities identified by experts were similarly broad in scope. It is our hope that as the sector progresses in the collection and translation of rigorous evidence that future priority setting exercises on child protection in humanitarian settings will be able to focus on particular needs within narrower sub-specialties.

### Limitations

The CHNRI method is based on purposive sampling where individuals are invited to participate based on their expertise in a given field. This method relies on a non-representative sample to aggregate knowledge and experiences. The findings are therefore limited to the perceptions of a discrete group of individuals and it is possible that additional areas for research investment may have emerged if a larger sample was recruited though, as earlier noted, prior quantitative work has demonstrated collective opinion to stabilize with as few as 45–55 participants [29], however, this finding was based on binary “yes” or “no” responses as opposed to the Likert scale implemented in this project. Further, given the low-cost and replicability of the procedure, it is attractive to a variety of sectors as a means of fostering transparency and enhancing systematization in the creation of a research agenda.

In our study. non-field based staff were more likely to respond to requests for interviews and as such, had greater representation within the study (Table 1). This created a certain level of bias towards the insights and experiences of child protection experts currently based in non-operational settings. When secondarily analyzing results based on whether respondents resided in operational or non-operational settings, we did find variation in the prioritization of research items (Table 4). These findings indicate that even when saturation appears to have been reached, the rank ordering of priorities can be influenced by the characteristics of the sample.

Deviating from standard CHNRI procedure, we requested that participants rank research priorities against pre-determined criteria using a Likert scale as opposed to binary “yes” or “no” responses. This decision was informed by the lack of existing evidence within the sector of child protection in humanitarian action and the anticipation that a large majority of research items would be affirmatively ranked by respondents, making it difficult to discern which were of highest priority. While Likert scales have been used extensively in other crowdsourcing methods [56–58], more research is needed to examine the benefits and drawbacks of using a Likert scale within an adapted CHNRI framework.

Lastly, our study did not include “impact” as a ranking criterion. Such a criterion would have participants rank research based on the likelihood it would result in a reduction of protection risks or improved responses to child protection violations. While our research criterion of “relevance” included similar language, it did not explicitly request input on the ability of a research question, once answered, to impact the lives of children. Further research priority setting exercises on child protection may wish to include “impact” as a ranking criterion separate from “relevance” in order to further ascertain the merit of a research idea.

## Conclusion

Rigorous, scientific research that assesses the scope of child protection risks, examines the effectiveness of existing child protection interventions, and translates evidence to practice is critical to move the sector forward and respond to donor calls for programming that is evidence-based. This CHNRI adaptation solicited inputs from a range of sector experts with variation across geographic location and job function. It is our hope that findings can guide a global research agenda, facilitating cooperation among donors, implementers, and academics to pursue a coordinated approach to evidence generation.

## Supporting information

S1 Annex Full ranking. (DOCX)

## Abbreviations

ACPHA: Alliance for Child Protection in Humanitarian Action
AEA: Average Expert Agreement
AME: Assessment, Measurement & Evaluation Working Group
CHNRI: Child Health and Nutrition Research Initiative
CP AoR: Child Protection Area of Responsibility
RPS: Research Priority Scores
UNHCR: United Nations High Commissioner for Refugees
NGO: Non-Governmental Organizations
UN: United Nations
UNICEF: United Nation Children’s Fund
