# Supplementary material for: Setting global research priorities for child protection in humanitarian action: Results from an adapted CHNRI exercise
Source: PLoS One. 2018 Aug 22;13(8):e0202570. doi: 10.1371/journal.pone.0202570 (PMC6104993; doi:10.1371/journal.pone.0202570)
Supplement: S1 Annex — (DOCX) [file pone.0202570.s001.docx]

| Overall Rank | Research Question | Research Type | Relevance | Feasibility | Originality | Applicability | Average Expert Agreement AEA | Research Priority Score RPS(SD) |
| --- | --- | --- | --- | --- | --- | --- | --- | --- |
| 1 | Rigorously evaluate the effectiveness of cash-based social safety nets to improve child well-being | INT | 90.29 | 87.27 | 80.00 | 87.74 | 85.63 | 86.33 (4.42) |
| 2 | Rigorously evaluate the effectiveness of family strengthening interventions to improve child well-being | INT | 89.44 | 83.03 | 79.35 | 82.94 | 83.09 | 83.69 (4.20) |
| 3 | Identify best practices for para-social work models in humanitarian settings | PSR | 86.45 | 84.67 | 77.93 | 82.67 | 78.21 | 82.93 (3.67) |
| 4 | Rigorously evaluate the effect of multi-sectoral programs on child well-being. Analyze how various components interact with one another. | INT | 86.06 | 78.62 | 85.16 | 81.29 | 83.63 | 82.78 (3.46) |
| 5 | Examine systems strengthening interventions to determine which have a measurable impact on children. | PSR | 89.47 | 79.44 | 77.22 | 83.33 | 77.27 | 82.37 (5.37) |
| 6 | Estimate the prevalence of child labor in humanitarian settings | EPI | 88.95 | 81.11 | 76.00 | 82.78 | 80.45 | 82.21 (5.34) |
| 7 | Identify best practices for bridging humanitarian and development initiatives for CP systems strengthening | PSR | 84.44 | 84.00 | 76.97 | 82.86 | 75.38 | 82.07 (3.46) |
| 8 | Rigorously evaluate the effectiveness of psychosocial programming to improve child well-being | INT | 88.33 | 86.06 | 70.00 | 82.35 | 78.02 | 81.69 (8.17) |
| 9 | Rigorously evaluate the added value of child protection interventions when mainstreamed within other sectors (i.e. health, education) | INT | 87.50 | 76.43 | 82.07 | 79.33 | 82.88 | 81.33 (4.71) |
| 10 | Rigorously evaluate the effectiveness of interventions to reduce child labor | INT | 86.47 | 81.94 | 76.13 | 80.63 | 85.75 | 81.29 (4.26) |
| 11 | Rigorously evaluate the effectiveness of capacity building interventions (for gov’t officials, social service workers, para social workers) to understand their impact on child well-being | PSR | 85.95 | 77.65 | 77.14 | 83.43 | 80.68 | 81.04 (4.34) |
| 12 | Identify risk factors associated with children with disabilities (with a particular emphasis on non-observable disabilities) | EPI | 84.12 | 80.63 | 78.06 | 81.29 | 81.91 | 81.02 (2.49) |
| 13 | Rigorously evaluate the effectiveness of case management to improve child well-being | INT | 86.47 | 81.82 | 75.76 | 80.00 | 73.62 | 81.01 (4.44) |
| 14 | Identify best practices for engaging the local social service workforce in emergency settings | PSR | 84.74 | 81.08 | 77.06 | 81.11 | 76.29 | 81.00 (3.14) |
| 15 | Translate existing literature on humanitarian emergencies in urban settings to better adapt CP program to current contexts | INT | 86.25 | 80.00 | 76.55 | 80.00 | 77.22 | 80.70 (4.04) |
| 16 | Rigorously evaluate the effectiveness of child protection committees to improve child well-being | INT | 85.56 | 84.24 | 72.35 | 80.59 | 78.75 | 80.68 (5.94) |
| 17 | Identify best practices for holistic/integrated case management in emergency contexts (such that one CM can address a range of needs within a household as opposed to being singularly focused on CP or GBV) | INT | 82.94 | 80.65 | 74.00 | 82.67 | 70.14 | 80.06 (4.17) |
| 18 | Rigorously evaluate the effectiveness of alternative care to improve the well-being of unaccompanied and separated children | INT | 84.85 | 81.33 | 73.79 | 80.00 | 72.82 | 79.99 (4.61) |
| 19 | Identify best practices for encouraging sustainable, long-lasting engagement on interventions such as child protection committees | INT | 82.76 | 80.71 | 72.59 | 81.43 | 74.79 | 79.37 (4.60) |
| 20 | Translate existing literature on broader care reform (ie foster care) to improve program design for alternative care in emergency settings | INT | 82.50 | 80.00 | 72.59 | 79.31 | 65.62 | 78.60 (4.23) |
| 21 | Examine the role of religious courts in child protection (child marriage, alternative care) | PSR | 81.76 | 76.25 | 77.42 | 78.13 | 71.94 | 78.39 (2.38) |
| 22 | Rigorously evaluate the effectiveness of interventions to reduce child marriage | INT | 81.18 | 79.35 | 75.33 | 77.42 | 75.16 | 78.32 (2.51) |
| 23 | Translate existing literature on adolescent development into program design | INT | 81.14 | 80.00 | 72.50 | 76.97 | 76.47 | 77.65 (3.86) |
| 24 | Build the capacity of the child protection sector staff in empirical research design and data analysis planning | INT | 81.71 | 76.47 | 75.15 | 77.06 | 69.04 | 77.60 (2.86) |
| 25 | Assess the added value of child participation in obtaining improved outcomes in child protection programming | INT | 84.12 | 77.33 | 72.50 | 75.63 | 71.69 | 77.39 (4.91) |
| 26 | Develop strategy to help programs identify if they are (or are not) reaching the most vulnerable (indigenous children, refugee children, migrant children, girls who have married early, girls separated from school, refugees not registered with UNHCR, etc). | INT | 82.42 | 76.00 | 71.33 | 78.71 | 69.84 | 77.12 (4.67) |
| 27 | Examine the common drivers and gender dynamics of child labor in humanitarian settings | EPI | 78.89 | 76.97 | 75.76 | 76.47 | 72.00 | 77.02 (1.34) |
| 28 | Identify best practices for serving inaccessible communities | INT | 81.18 | 72.50 | 74.84 | 79.38 | 74.24 | 76.97 (4.00) |
| 29 | Translate existing literature on social-ecological models into program design | INT | 81.33 | 77.04 | 74.62 | 74.29 | 63.74 | 76.82 (3.25) |
| 30 | Assess whether the INSPIRE framework is relevant within fragile contexts | INT | 76.36 | 76.84 | 77.00 | 77.00 | 71.72 | 76.80 (0.30) |
| 31 | Identify good practices to serve LGBTQI adolescents in contexts with severe protection considerations | INT | 81.48 | 72.50 | 80.91 | 72.31 | 73.69 | 76.80 (5.08) |
| 32 | Identify best practices for transfer/return of adolescent victims of trafficking that do not place them at risk of harm | PSR | 83.75 | 76.67 | 71.85 | 74.67 | 66.65 | 76.73 (5.08) |
| 33 | Develop guidance on how the CP sector can use data to make real-time adjustments/adaptations to their programs for continuous improvement | INT | 81.71 | 73.55 | 76.36 | 75.00 | 73.68 | 76.66 (3.56) |
| 34 | Estimate the prevalence of sexual violence against children in humanitarian settings (with improved data collection on boys) | EPI | 85.26 | 70.00 | 72.00 | 78.33 | 67.25 | 76.40 (6.89) |
| 35 | Translate existing literature on sexual violence against boys to improve program design. | INT | 81.71 | 75.00 | 73.55 | 75.15 | 63.80 | 76.35 (3.65) |
| 36 | Examine if targeted trainings for caregivers are a practical and effective way for treating children with developmental/intellectual disabilities | INT | 77.78 | 76.00 | 75.83 | 75.00 | 64.84 | 76.15 (1.17) |
| 37 | Estimate the prevalence of children in alternative care in humanitarian settings | EPI | 78.86 | 77.06 | 69.41 | 78.82 | 71.51 | 76.04 (4.50) |
| 38 | Translate existing literature on early childhood development (attachment, adversities, toxic stress, etc) into program design | INT | 79.35 | 78.57 | 72.31 | 73.79 | 70.76 | 76.01 (3.48) |
| 39 | Assess the drivers of transactional/survival sex among adolescent girls | EPI | 81.18 | 75.00 | 69.68 | 78.13 | 67.89 | 75.99 (4.91) |
| 40 | Estimate the prevalence of emotional disturbances and self-injurious behaviors among unaccompanied migrant adolescents (self-mutiliation, substance abuse, severe depression) | EPI | 81.67 | 69.09 | 78.82 | 74.38 | 66.42 | 75.99 (5.49) |
| 41 | Identify best practices for improving social cohesion at the community level (both between migrant and host communities and within migrant communities) | PSR | 79.49 | 78.89 | 69.19 | 76.22 | 69.12 | 75.95 (4.72) |
| 42 | Estimate the prevalence of trafficked children in humanitarian settings | EPI | 87.22 | 65.29 | 73.13 | 77.65 | 66.52 | 75.82 (9.15) |
| 43 | Identify best practices in Information Management Systems (how to be more efficient, less cumbersome to field staff, and produce usable data for analysis) | INT | 80.59 | 77.50 | 70.00 | 75.00 | 68.34 | 75.77 (4.48) |
| 44 | Mainstream child protection indicators within the data collection protocols of other sectors | INT | 82.22 | 74.71 | 67.27 | 77.58 | 65.10 | 75.44 (6.27) |
| 45 | Estimate the prevalence of child labor among host communities affected by displacement | EPI | 78.89 | 76.00 | 74.29 | 72.57 | 66.63 | 75.44 (2.69) |
| 46 | Rigorously evaluate the effectiveness of psychological first aid | INT | 78.86 | 74.38 | 71.88 | 76.25 | 65.51 | 75.34 (2.95) |
| 47 | Identify the risk factors associated with recruitment of children and adolescents to armed forces and armed groups (updated for current crises – al shabab, ISIS, etc) | EPI | 84.12 | 74.38 | 65.00 | 77.50 | 63.56 | 75.25 (7.95) |
| 48 | Estimate the prevalence of violence against children in schools in humanitarian settings | EPI | 77.22 | 78.29 | 66.86 | 77.71 | 62.38 | 75.02 (5.46) |
| 49 | Identify best practices for improving birth registration in emergency settings | INT | 77.58 | 74.84 | 70.67 | 76.77 | 70.23 | 74.96 (3.09) |
| 50 | Identify good practices for serving children associated with armed forces and armed groups (updated for current contexts) | INT | 80.00 | 74.29 | 70.00 | 75.00 | 68.35 | 74.82 (4.10) |
| 51 | Identify best practices on establishing functioning referral networks in zones of armed conflict | PSR | 82.35 | 72.12 | 70.63 | 73.94 | 59.67 | 74.76 (5.24) |
| 52 | Estimate the prevalence of violence against children in the home in humanitarian settings | EPI | 82.86 | 72.57 | 66.47 | 77.06 | 63.68 | 74.74 (6.94) |
| 53 | Translate existing literature on disaster risk reduction and child protection into program design within the humanitarian sector | INT | 76.97 | 76.00 | 69.66 | 76.13 | 72.17 | 74.69 (3.38) |
| 54 | Use predictive analytics to identify common determinants/risk factors across typical child protection issues at the population level | EPI | 77.65 | 72.50 | 75.63 | 72.50 | 68.38 | 74.57 (2.53) |
| 55 | Identify best practices for working with non-state actors for child protection in humanitarian settings | PSR | 81.08 | 72.00 | 68.57 | 76.00 | 65.27 | 74.41 (5.38) |
| 56 | Rigorously evaluate the effectiveness of interventions to reduce the physical punishment of children | INT | 78.29 | 76.36 | 68.00 | 74.55 | 68.23 | 74.30 (4.47) |
| 57 | Estimate the prevalence of early marriage in humanitarian settings | EPI | 81.08 | 71.76 | 69.14 | 74.44 | 79.55 | 74.11 (5.13) |
| 58 | Identify best practices for operationalizing CP within the PSS pyramid | INT | 76.77 | 75.17 | 70.34 | 73.79 | 61.76 | 74.02 (2.74) |
| 59 | Conduct a review of potential methodologies that can be used to support evidence generation in emergency settings | INT | 79.41 | 76.25 | 68.75 | 71.52 | 67.01 | 73.98 (4.76) |
| 60 | Rigorously evaluate the effectiveness of awareness raising approaches to facilitate behavior/social norms change on child protection issues | INT | 79.44 | 72.94 | 67.27 | 75.29 | 67.60 | 73.74 (5.08) |
| 61 | Define terminology such as “child well-being”, “life-saving”, “quality responses” in order to systematically and quantitatively measure outcomes and compare across regions | INT | 78.33 | 71.76 | 69.41 | 74.71 | 57.11 | 73.55 (3.85) |
| 62 | Develop a more systematic approach to localization and cultural adaption of evidence to improve program design and implementation | INT | 78.13 | 71.33 | 72.00 | 72.67 | 61.30 | 73.53 (3.11) |
| 63 | Evaluate the quality of coordination at the field level (i.e if minimum standards are being applied) and its effect on child well-being outcomes | INT | 76.36 | 69.38 | 70.97 | 71.88 | 63.19 | 72.15 (3.00) |
| 64 | Compare risks and vulnerabilities for children living in refugee, IDP, and non-refugee settings to determine if interventions should be modified depending on the context | EPI | 74.12 | 75.63 | 66.88 | 71.88 | 63.79 | 72.12 (3.82) |
| 65 | Identify the risks that climate change pose for child protection globally and regionally | EPI | 75.63 | 67.86 | 74.81 | 68.67 | 61.56 | 71.74 (4.04) |
| 66 | Estimate the prevalence of separated children in humanitarian settings | EPI | 76.22 | 76.00 | 61.18 | 73.14 | 57.92 | 71.63 (7.11) |
| 67 | Develop a child protection vulnerability assessment at the household level | INT | 75.15 | 73.33 | 65.63 | 72.26 | 60.30 | 71.59 (4.15) |
| 68 | Identify best practices for jointly conceptualizing CP and SGBV within interventions and measurement | INT | 73.94 | 70.97 | 69.03 | 71.61 | 53.05 | 71.39 (2.02) |
| 69 | Develop case studies on aid-induced harm | INT | 73.55 | 71.72 | 66.90 | 73.10 | 57.51 | 71.32 |
| 70 | Estimate the prevalence of detained children in humanitarian settings | EPI | 79.47 | 65.14 | 68.33 | 70.29 | 55.98 | 70.81 (6.15) |
| 71 | Analyze the emergency legal processing systems instituted in Europe to address the migrant crises and their effects on migrant children (particularly those that are separated or unaccompanied) | PSR | 73.85 | 74.17 | 66.09 | 68.33 | 59.39 | 70.61 (4.03) |
| 72 | Rigorously evaluate the effectiveness of interventions to protect children on the move | INT | 76.36 | 64.67 | 69.03 | 72.26 | 60.49 | 70.58 (4.95) |
| 73 | Incorporate cost-effectiveness and cost-benefit analysis into research frameworks | INT | 74.19 | 70.37 | 66.00 | 69.03 | 54.56 | 69.90 (3.40) |
| 74 | Define "resilience" and identify those factors that build the resilience of children and families | EPI | 73.89 | 72.35 | 60.00 | 72.94 | 60.51 | 69.80 (6.56) |
| 75 | Rigorously evaluate the effectiveness of child friendly spaces to improve child well-being | INT | 75.00 | 74.71 | 56.47 | 71.76 | 65.85 | 69.49 (8.80) |
| 76 | Modify situational analysis tools so that results better feed into broader evidence building for the sector | INT | 71.61 | 71.03 | 66.21 | 68.97 | 54.12 | 69.45 (2.45) |
| 77 | Identify best practices for operational organizations to support agency-based intervention research | INT | 70.71 | 72.31 | 66.15 | 68.46 | 51.85 | 69.41 (2.68) |
| 78 | Examine the changing dynamics of conflict settings and how organizations can adapt accordingly | INT | 73.13 | 66.67 | 65.33 | 68.00 | 58.02 | 68.28 (3.41) |
| 79 | Conduct a secondary review of literature on all CP areas of concern in order to have tangible data available for funding appeals and proposals | INT | 70.56 | 72.35 | 58.18 | 70.00 | 53.82 | 67.77 (6.47) |
| 80 | Evaluate how investment in girls also achieves improved outcomes for boys, families, and communities | INT | 68.13 | 67.59 | 66.90 | 66.90 | 49.57 | 67.38 (0.60) |
| 81 | Conduct a meta-analysis on research involving adolescent girls in humanitarian contexts | INT | 71.61 | 70.34 | 60.00 | 66.90 | 46.41 | 67.21 (5.21) |
| 82 | Identify best practices for harnessing technology to improve accountability | INT | 70.00 | 68.13 | 63.87 | 65.16 | 43.60 | 66.79 (2.78) |
| 83 | Estimate the prevalence of displaced children in humanitarian settings | EPI | 70.27 | 72.00 | 56.57 | 67.06 | 53.19 | 66.48 (6.91) |
| 84 | Conduct feasibility study on family tracing and reunification in Syria |  | 70.40 | 66.09 | 63.33 | 65.83 | 40.53 | 66.41 (2.93) |
| 85 | Assess durable solutions in Syria and what support will be needed to ensure proper protection and reintegration for children | INT | 71.72 | 61.54 | 64.62 | 66.92 | 42.74 | 66.20 (4.29) |
| 86 | Conduct a review of toolkits/operational guides at the field level to assess practicality and applicability for local settings, determine what can be condensed, what new guidance is needed | INT | 68.48 | 67.74 | 60.65 | 67.74 | 53.91 | 66.15 (3.69) |
| 87 | Rigorously evaluate the effectiveness of app-based approaches to psychosocial support with children on the move | INT | 67.62 | 61.05 | 70.53 | 62.11 | 47.31 | 65.33 (4.51) |
| 88 | Examine the cluster system and identify if there are duplications and challenges around coordination and operationalization | INT | 66.67 | 67.74 | 56.77 | 65.81 | 46.80 | 64.25 (5.04) |
| 89 | Identify the risk factors for substance use among adolescent males | EPI | 70.91 | 65.16 | 56.67 | 63.23 | 44.53 | 63.99 (5.87) |
| 90 | Estimate the prevalence of female genital mutilation/cutting in humanitarian settings | EPI | 65.71 | 61.88 | 62.42 | 63.13 | 41.55 | 63.28 (1.70) |
